# Tripling the light extraction efficiency of a deep ultraviolet LED using a nanostructured p-contact

**DOI:** 10.1038/s41598-022-15499-7

**Published:** 2022-07-07

**Authors:** Eduardo López-Fraguas, Felix Binkowski, Sven Burger, Sylvia Hagedorn, Braulio García-Cámara, Ricardo Vergaz, Christiane Becker, Phillip Manley

**Affiliations:** 1 GDAF-UC3M, Dep. Tecnología Electrónica, Universidad Carlos III de Madrid, Avda. Universidad, 30., 28911 Leganés, Madrid Spain; 2 Zuse Institute Berlin, Berlin, Takustraße 7, 14195 Berlin, Germany; 3 JCMwave GmbH, Bolivarallee 22, 14050 Berlin, Germany; 4 Ferdinand-Braun-Institut (FBH), Gustav-Kirchhoff-Str. 4, 12489 Berlin, Germany; 5 Department Optics for Solar Energy, Helmholtz Zentrum Berlin für Materialen und Energie, Kekuléstr. 5, 12489 Berlin, Germany

**Keywords:** Inorganic LEDs, Optoelectronic devices and components

## Abstract

Despite a wide array of applications, deep ultra-violet light emitting diodes offer relatively poor efficiencies compared to their optical counterparts. A contributing factor is the lower light extraction efficiency due to both highly absorbing p-contacts and total internal reflection. Here, we propose a structure consisting of a hexagonal periodic array of cylindrical nanoholes in the multi-layered p-contact which are filled with platinum. This nanostructure reduces the absorption of the p-contact layer, leading to a higher emission into the n-contact compared to a planar reference. An optimum geometry of the nanostructure allows a light extraction efficiency of 15.0%, much higher than the typical 4.6% of a planar reference. While the nanostructure strongly decreases the light absorption in the p-contact, it is still not able to considerably reduce the total internal reflection. Consequently, the nanostructured p-contact should be combined with other optical strategies, such as nanopatterned sapphire substrates to increase the efficiency even further. Despite this, the nanostructure described in this work provides a readily realizable path to enhancing the light extraction efficiency of state-of-the-art deep ultra-violet light emitting diodes.

## Introduction

Light emitting diodes (LEDs) in the visible are contributing towards the technological green revolution^1^. The energy efficiency of LEDs can be exploited for other applications by shifting the emission wavelength. Deep ultra-violet light emitting diodes (DUV-LEDs)^2^ have generated great interest due to their numerous applications^3^ such as: water purification^4^, 3D printing and UV curing^5^, sterilization^6,7^, food treatment^8^, sensing^9^ and even security applications^10^.

**Figure 1:**
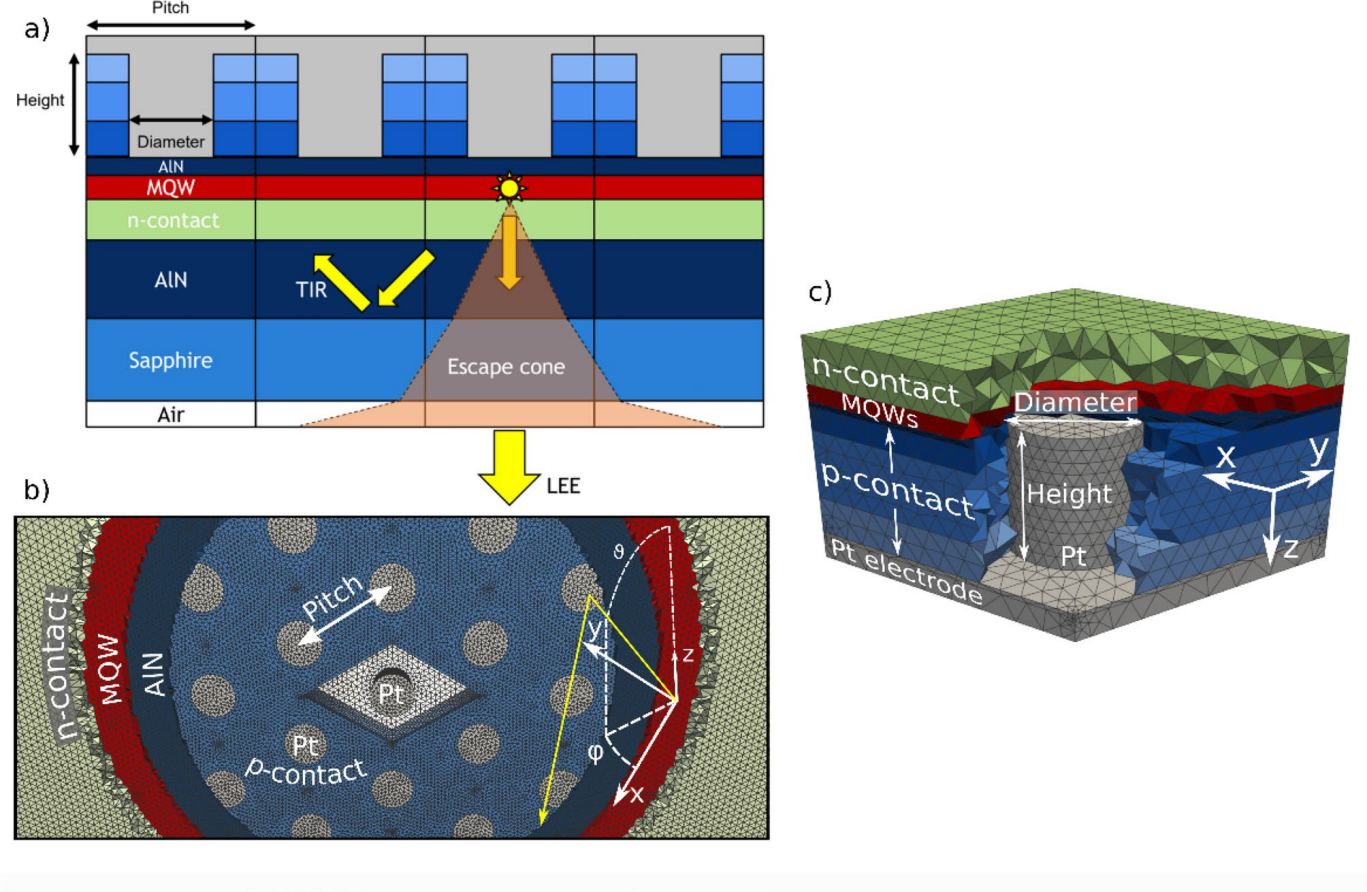
(**a**) Cross-section schematic of the nanostructured device showing the escape cone and total internal reflection (TIR). (**b**) The nanostructured device showing the central unit cell of the periodic lattice (diamond shape) with the p-contact, which completely fills the space between nanocylinders, hidden for visibility. The AlN, multiple quantum wells (MQW) and n-contact layers have also been partially obscured for visibility. (**c**) The periodic unit cell of the nanocylinder with layers partially hidden for visibility.

The promise of DUV-LEDs can only be fulfilled if light can be efficiently extracted, which can be quantified by the light extraction efficiency (LEE)^11^. One hurdle towards a high LEE that all LEDs must overcome is total internal reflection (TIR). This describes light being trapped inside a device due to having a higher refractive index than the surrounding media. A schematic representation of TIR is shown in Fig. 1a. To counteract TIR, a fitting concept must be implemented to redirect light into the escape cone, also pictured in Fig. 1a, meaning that it can leave the device. Many such concepts have been developed for LEDs, among them are rough surfaces^12,13^, micro-lenses^14,15^, plasmonic gratings^16^, dielectric nanoparticles^17,18^, bio-mimetic structures^19^, nanophotonic structures^20^, photonic crystals^21–23^ and nanopatterned substrates^24^.

A second challenge for DUV-LEDs, is that the standard materials used for the p-contact are highly absorbing^25^. Therefore, at least half of the emitted light will be lost due to absorption in the p-contact. Attempts to replace the highly absorbing contact with lower absorption materials constitutes an active research topic^26–28^. However, it is currently unclear if the voltage and lifetimes of these alternative contacts can stay within acceptable limits^5^.

Absorption in the p-contact may also be reduced via nanostructuring. This can reduce both the absorption, thereby increasing the reflection of the p-contact, and reduce TIR by scattering light into the escape cone. Previous work has studied the effect of creating nanoholes in the metallic electrode^27^ or nanovoids in the p-contact itself^29^. Instead, here we propose periodically arranged nanocylinders of the electrode which are embedded in the p-contact. It has been shown that this setup can efficiently couple transverse travelling modes to external radiation, thus scattering trapped light towards the exterior^30^. Due to the multifaceted solution to the problems of DUV-LEDs that nanostructured p-contacts provide, their potential use needs to be carefully analyzed.

This paper is focused on the enhancement of the optical properties of DUV-LEDs including a periodic nanostructured p-contact. However, nanostructuring the contact can undermine the electrical quality of the p-contact itself. This can be further exacerbated by using highly reflective materials for the electrode such as Al^27^ which further increases the contact resistance. To avoid this, we seek nanostructures that can form a large area ohmic contact to the p-type part of the DUV-LED heterostructure for efficient carrier injection. Typical contact materials used to achieve low resistance p-type ohmic contacts are palladium (Pd) and platinum (Pt)^5^. In this work we employ Pt as the contact material which has a reflectivity of 56% at 265 nm in air and at normal incidence. Previous work has tended to focus on Al as a contact material with a reflectivity of 93% at 265 nm in air and at normal incidence, but a corresponding higher contact resistance. At the same time, the nanostructured contacts should penetrate deeply through the non-transparent part of the p-side to reduce the optical absorption. Therefore, although being focused on the optical response, this work provides guidelines for obtaining an optimal tradeoff between electrical and optical performance.

In the following sections we first describe the DUV-LED system with an integrated nanostructure. This is followed by a detailed report of the numerical methods employed, including the method by which the periodic nanostructure can be accounted for while keeping a quasi-isolated dipole source. To this end we employ a method combining Brillouin zone integration and spatial integration in the multiple quantum well (MQW) layer to obtain a solution that approximates an ensemble of randomly-distributed isolated dipoles emitting light in a periodically structured environment. Having developed the model, we then apply it to a periodic system of platinum nanocylinders embedded in the p-contact. The emission of the device into both the n-contact and the exterior are analyzed separately. The geometrical parameters of the nanostructure are then varied and their effect on the LEE is analyzed, including a detailed look at the angular distribution of the emission into the n-contact. Furthermore, the ability of the nanostructure to scatter trapped light into the escape cone of the device is assessed. This allows the proposed nanostructure to be evaluated for both the aspects of reduced p-contact absorption and a reduction in TIR.

## Model and numerical methods

To model the light emission from the MQWs and the eventual LEE, we solve Maxwell’s equations for the light propagation inside the layered and nanostructured system of materials. The light is emitted by a dipole source inside the MQW layer, and the fraction of this emission which is able to escape the device into air provides an estimation of the LEE. In the following, we describe the different aspects of the design and simulations in more detail.

### Device composition

The nanostructured device, shown in Fig. 1a, consists of a platinum (Pt) cylinder that extends from the Pt superstrate through one or more layers of the multi-layer p-contact. Below the lower edge of the cylinder the device continues as a planar multi-layer stack. The cylinders are periodically arranged in a hexagonal lattice. Figure 1b shows the periodicity of the nanostructure as well as the unit cell, with certain layers partially removed to better visualize the cylindrical nanostructure. The nanostructure is fully described by the cylinder diameter, cylinder height and the pitch of hexagonal lattice of cylinders shown in Fig. 1b,c. The aspect ratio of the nanocylinder is given by the ratio of height to diameter, $AR=H/D$.

The DUV-LED multi-layer stack used as a reference in this work can be found in Refs.^31–33^, as well as their corresponding refractive indices and dimensions. The planar layers, from top to bottom, are: 30 nm of Pt contact (electrode), 40 nm of GaN:Mg, 75 nm of Al_0.285_Ga_0.715_N:Mg, 25 nm of Al_0.75_Ga_0.25_N:Mg (these three layers form the p-contact), 6 nm of AlN (electron blocking layer), 18 nm of multiple quantum well (MQW) layers that alternate between Al_0.47_Ga_0.53_N:Si (1 nm) as an emitter and Al_0.6_Ga_0.4_N:Si (5 nm) as a barrier to act as carrier confinement (these constitute the active layer), 1000 nm of Al_0.9_Ga_0.1_N:Si (the n-contact), 4 µm of AlN and a 400 µm sapphire substrate. This information is summarized, along with the associated complex refractive index of each material, in Table 1. We note that AlN has been used as an electron blocking layer due to improved suppression of parasitic luminescence compared to Al(Ga)N:Mg^35,36^.

**Table 1:** The materials used for the simulated devices, including their refractive index and absorption coefficient at 265 nm wavelength, and thicknesses in the planar reference device. Layer thicknesses and material compositions were taken from^31–33^, for Al_0.75_Ga_0.25_ N:Mg a linear interpolation scheme was used to obtain both refractive index and absorption coefficient^34^.

Material	Layer thickness	Refractive index	Absorption coefficient (cm^−1^)
Pt	30 nm	1.1653 + *i*2.4307	$1.153\times {10}^{6}$
GaN:Mg	40 nm	2.6981 + *i*0.476	$2.257\times {10}^{5}$
Al_0.285_Ga_0.715_N:Mg	75 nm	2.6103 + *i*0.340	$1.612\times {10}^{5}$
Al_0.75_Ga_0.25_N:Mg	25 nm	2.4774 + *i*0.119	$5.643\times {10}^{4}$
Multiple quantum well	18 nm	2.9532	–
Al_0.9_Ga_0.1_N:Si	1 µm	2.3723	–
AlN	4 µm	2.3147	–
Sapphire	400 µm	1.8337	–

### Nanooptical model

The nanostructure employed in the p-contact is periodic up to the boundary of the device. Therefore, we model only the periodic unit cell and apply periodic boundary conditions in the lateral directions with transparent boundary conditions in the vertical directions. This corresponds to a chip that is effectively infinitely extended in the *x–y* plane. This method is valid when the emission of light occurs far from the physical boundaries of the device and material absorption stops light from propagating a long distance in the *x–y* plane. The light emitted from a MQW can be well approximated via a dipole source, with an arbitrary position (inside the MQW) and an arbitrary phase. Unfortunately, by applying periodic boundary conditions, a dipole source inside the computational domain will also have its phase replicated, resulting in a periodic array of identical dipole sources emitting light coherently. To avoid this, previous work has tended to rely on computing large super-cells in the lateral direction^27,29,37^. Instead, in this work we employ a method of Brillouin zone integration to obtain a solution approximating an isolated dipole in a periodic device, without the need to calculate large supercells^38,39^. This has the benefit of converting a single simulation with a large computational domain into many simulations on computational domains with a much smaller volume.

The sources were modeled using a periodic dipole with an emission wavelength of 265 nm. As previously stated, applying a periodic dipole source leads to artificial, unrealistic interference effects as, in reality, the sources do not emit coherently with one another. In order to circumvent this, the integration over the phase of the periodic dipole source can be performed. With this, it is possible to obtain the electric field ${\text{E}}({\text{r}})$ resulting from an isolated dipole source ${\text{J}}({\text{r}})$ with an integration of Bloch-periodic electric field solutions,

$${\text{E}}\left({\text{r}}\right)=\frac{1}{\left|{\text{BZ}}\right|}\int _{\text{BZ}}{{\text{E}}}_{{\text{k}}_{\text{B}}}\left({\text{r}}\right)d{\text{k}}_{\text{B}},$$

where ${\text{BZ}}=\left\{{\text{k}}_{\text{B}}={\text{b}}\cdot \tau |\tau \in \left[\mathrm{0,1}\right]\times \left[\mathrm{0,1}\right]\right\}$ is the Brillouin zone defined by $${\left[{\text{b}}_{1}{,{\text{b}}}_{2}\right]}^{\text{T}}\cdot \left[{\text{a}}_{1}{,{\text{a}}}_{2}\right]=2\pi \left[\begin{array}{cc}1& 0\\ 0& 1\end{array}\right]$$. The matrix $\text{a} = \left[{\text{a}}_{1}{,{\text{a}}}_{2}\right]\in$ ${\mathbb{N}}^{2\times 2}$ contains the lattice vectors of the underlying periodic structure. The lattice vectors are two-dimensional due to the twofold periodicity. The Bloch-periodic fields ${\text{E}}_{{\text{k}}_{\text{B}}}\left({\text{r}}\right)$ are the solutions to Maxwell’s equations with Bloch-periodic dipole sources ${\text{J}}_{{\text{k}}_{\text{B}}}\left({\text{r}}\right)$, for different phase vectors ${\text{k}}_{\text{B}}$. This approach is based on the Floquet transform ${\text{J}}_{{\text{k}}_{\text{B}}}\left({\text{r}}\right)=\sum_{{\text{l}}\in {\mathbb{N}}^{2}}{e}^{i{\text{k}}_{\text{B}}^{\text{T}}{\text{a}}\cdot {\text{l}}}{\text{J}}({\text{r}}-{\text{a}}{\cdot}{\text{l}})$ and on the corresponding inverse Floquet transform ${\text{J}}({\text{r}})\boldsymbol{ }=\frac{1}{|{\text{BZ}}|}{\int }_{\text{BZ}}{{\text{J}}}_{{\text{k}}_{\text{B}}}\left({\text{r}}\right)d{\text{k}}_{\text{B}}$^38^. To numerically realize the integral over the Brillouin zone, we applied a trapezoidal rule with 16 Bloch-vectors ${\text{k}}_{\text{B}}$. Note that this leads to an approximation of the integral. An increase of the integration points is comparable to using supercells with Bloch-periodic sources with increasing size. Such a supercell is shown in Fig. 1b. The integrated values of absorption in each layer and light emission into the n-contact were shown to converge when using increasing numbers of Bloch vectors via a convergence study. Using 16 Bloch-vectors, the numerical error on these values was estimated to be already less than 1×10^−3^. Supplementary Figure S1 shows the suppression of interference between neighboring dipoles by employing Brillouin Zone integration.

Due to the stochastic spatial distribution of dipole sources in the MQW layer, the dipole position was also integrated using the trapezoidal rule to approximate the average dipole position. We used 64 positions on a regular grid in the *x–y* plane at the vertical position centered in the MQW layer.

The polarization of the emitting dipoles will contribute heavily to the LEE. In the literature emission from AlGaN based MQWs has been shown to transition from majority TE polarized light to TM polarized for wavelengths under 265 nm^40^. Due to the 265 nm emission wavelength of the dipole in the present work, we assume that the emission is TE polarized. Supplementary Figure S2 shows the dependence of the TM LEE on the nanocylinder height, confirming that the TM LEE remains much smaller than the TE LEE even in the presence of the nanostructure.

The light leaving the computational domain into the extended n-contact was decomposed into plane waves. This allows the modes to be categorized by their transverse wave vector components (*k*_x_, *k*_y_). Due to the periodic boundary conditions, only a discrete set of transverse wave vector components are excited. These depend on the shape and pitch of the unit cell.

The decomposition of the light emitted into the n-contact into plane waves allows for the propagation through the n-contact, the AlN layer and the sapphire substrate to be handled analytically. This is especially important for the current model due to the fact that those layers are many hundreds or even thousands of wavelengths thick, which is computationally very demanding for a rigorous method. Therefore, an analytical propagation of plane waves is significantly more efficient than a FEM solution in these domains.

Using Snell’s law, we can calculate which angles of light emission will be incapable of escaping the device due to TIR. The critical angle to the device normal (*z* direction) for light initially propagating in the n-contact to escape the sapphire/air boundary is 25°. Light with a propagation angle higher than this one will suffer from TIR.

### Numerical method

To take into account the diffractive effects of the nanostructure, a rigorous solver of Maxwell’s equations is mandatory. Nanostructured geometries incorporate a highly absorbing metallic region with both well-defined and smooth edges. The finite element method (FEM) is a well-suited numerical method for obtaining a solution in this case. Due to the freedom of locally refined and unstructured meshes, and locally refined polynomial degrees, FEM is able to efficiently model the interaction of light with both dielectrics and metals^41^. Previous works optically modeling light emission in DUV-LEDs have mainly employed the finite difference time domain (FDTD) method for solving Maxwell’s equations^27,29,37,42^. Due to the regular grid used in finite-difference time domain (FDTD), a higher computation effort is required to approximate the curved surfaces of the cylindrical nanostructure compared to FEM. We use the commercial software package JCMsuite, a rigorous solver for Maxwell’s equations employing FEM^43^. We note that the proposed model which relies on Brillouin Zone integration instead of calculating large supercells does not require FEM and could be employed using other numerical methods to solve Maxwell’s equations, like the Finite Difference Time Domain method (FDTD) or Rigorous Coupled Wave Analysis (RCWA).

As shown in Fig. 1b, the periodic nanostructure can be divided into a lattice of unit cells. Constricting the volume of the computation to a unit cell reduces the computational costs. Furthermore, to optimize the computational requirements, the model for light propagation can be divided into regions where a general solution to Maxwell’s equations is required (in the near field of the nanostructure) and regions in which plane wave propagation analysis is sufficient (the planar stack consisting of the n-contact, AlN and sapphire layers). The transparent boundary conditions in the vertical (*z*) direction of the unit cell were realized using perfectly matched layers (PML). The mesh element lengths were locally set in each material to ensure that they were not longer than a quarter of the local wavelength or skin depth (for dielectric and metals respectively) in the material. This results in a mesh with side lengths between 15 and 60 nm. The finite element degree was selected locally on each element using an a-priori estimator with a goal precision of 1×10^−4^. This results in typical values for the finite element degree between 3 and 5. The numerical error of the absorption and light emission into the n-contact was estimated for the reference structure to be smaller than 5×10^−3^ via a convergence study. Tabulated refractive index data for the various materials were taken from literature^31–33^ and are presented in Table 1. Due to the purely optical simulations, no assumptions are made regarding the IV parameters of the device.

## Results and discussion

The absorption in both the Pt electrode and p-contact layer, as well as the amount of light lost due to TIR, and the LEE, are shown in Table 2. These results represent three cases: a planar reference and the nanostructured LED with two different nanocylinder heights. The nanocylinder heights descript a high aspect ratio (140 nm) and a low aspect ratio (20 nm) structure, both with a 300 nm pitch and 150 nm diameter. The 140 nm height corresponds to the highest overall LEE seen in this study, while the 20 nm structure represents the highest LEE available while keeping the nanostructure height less than or equal to 20 nm.

**Table 2:** The absorption in the Pt electrode (Abs_Pt_) and p-contact region (Abs_P-Contact_), the light emission lost due to total internal reflection (TIR Losses), the light extraction efficiency (LEE) and ratio of LEE to TIR losses for the planar reference and nanostructured devices with 300 nm pitch and 150 nm diameter size.

	Abs_Pt_ (%)	Abs_P-Contact_ (%)	TIR Losses (%)	LEE (%)	LEE/TIR
Planar reference	0.9	67.3	27.2	4.6	0.17
Nanostructured (height 140 nm)	13.1	33.7	42.1	11.1	0.26
Nanostructured (height 20 nm)	1.0	51.0	40.0	8.0	0.20

Comparing the high aspect ratio nanostructure with a height of 140 nm with the planar case, we see that the absorption in the p-contact reduces from 67.3% to only 33.7%. However, the absorption in the Pt electrode increases to 13.1%. The net effect is that 53.2% of the emission is transmitted into the n-contact, compared with the 31.8% in the planar case. However, the amount of light that suffers from TIR increases to 42.1%. Overall, this means that the light extraction efficiency improves from 4.6 to 11.1%. In the final column of Table 2, we compare the ratio of between the amount of light that escapes (LEE) to that which undergoes TIR. Here we see that, although the nanostructure has overall higher TIR losses, the ratio of LEE to TIR is increased compared to the planar structure. This supports the idea that the nanostructure is able to scatter light emitted from the dipole source into the escape cone. Therefore, the net effect is that the nanostructured p-contact is able to greatly reduce the initial absorption in the p-contact and partially redirect the light into the escape cone, but still a large fraction of the light emission undergoes TIR and is absorbed upon a second interaction with the p-contact. This means that the LEE is almost tripled in this configuration, but it could be improved much further with a proper TIR reduction.

As stated above, we have also considered a low aspect ratio structure. Due to the distance to the active layer, this is a more realistic structure in terms of fabrication. Moreover, such a shallow nanostructure is likely to have less deleterious effects on the electrical performance compared to the high aspect ratio structure. This low aspect ratio structure shows a more moderate decrease in the p-contact absorption, reduced from 67.3 to 51%, when going from the planar to the nanostructured case. Despite this, similar amounts of light are lost due to TIR when compared to the high aspect ratio device. Additionally, the absorption in the Pt contact itself it limited to 1% for this shallow nanostructure. This validates the choice of Pt as a contact material for systems seeking a balance between increased LEE and preserving high electrical efficiency. This results in a LEE that is increased from 4.6 to 8.0% compared to the planar case. This result suggests that even a relatively shallow nanostructuring of the p-contact can still have a large impact on the LEE of the device.

In order to determine how the geometrical parameters of the nanostructure affect both the p-contact absorption and the light extraction, we varied the geometrical parameters along the following ranges:

- diameter from 50 to 250 nm, height and pitch being constant.
- height from 20 to 140 nm, diameter and pitch being constant.
- pitch from 200 to 450 nm, while height remains constant and ratio of diameter to pitch is one half.

**Figure 2:**
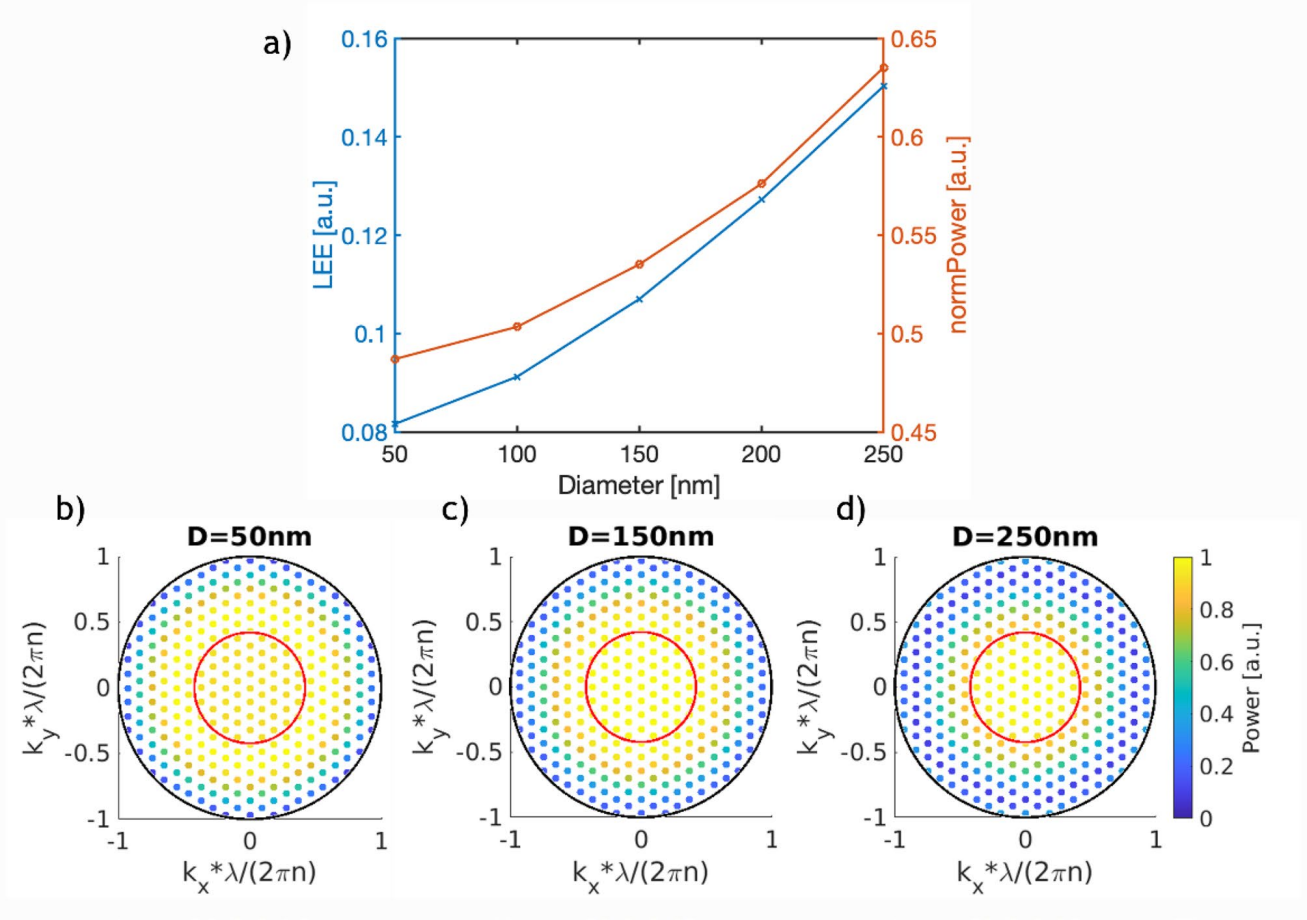
(**a**) LEE (blue) and normalized power emitted into the substrate (orange) as a function of the nanostructure diameter. (**b**–**d**) Normalized power distribution of modes emitted into the substrate for a diameter equal to 50 nm, 150 nm and 250 nm. The black outer circle represents the boundary of propagating modes, while the red inner circle represents the escape cone limited by TIR.

**Figure 3:**
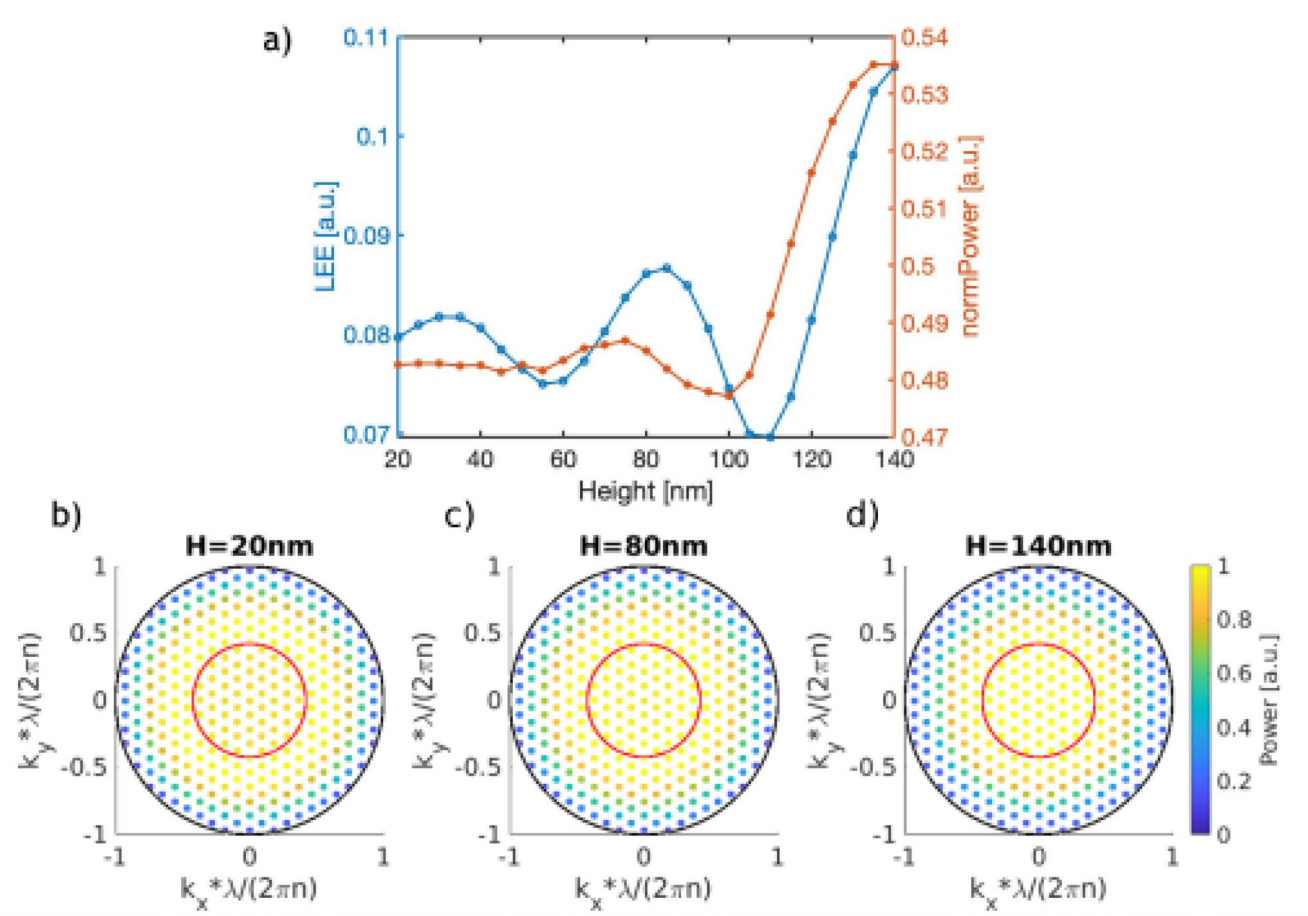
(**a**) LEE and normalized power emitted into the substrate as a function of the nanostructure height. (**b**–**d**) Normalized power distribution of modes emitted into the substrate for a height equal to 20 nm, 80 nm and 140 nm. The black outer circle represents the boundary of propagating modes, while the red inner circle represents the escape cone limited by TIR.

**Figure 4:**
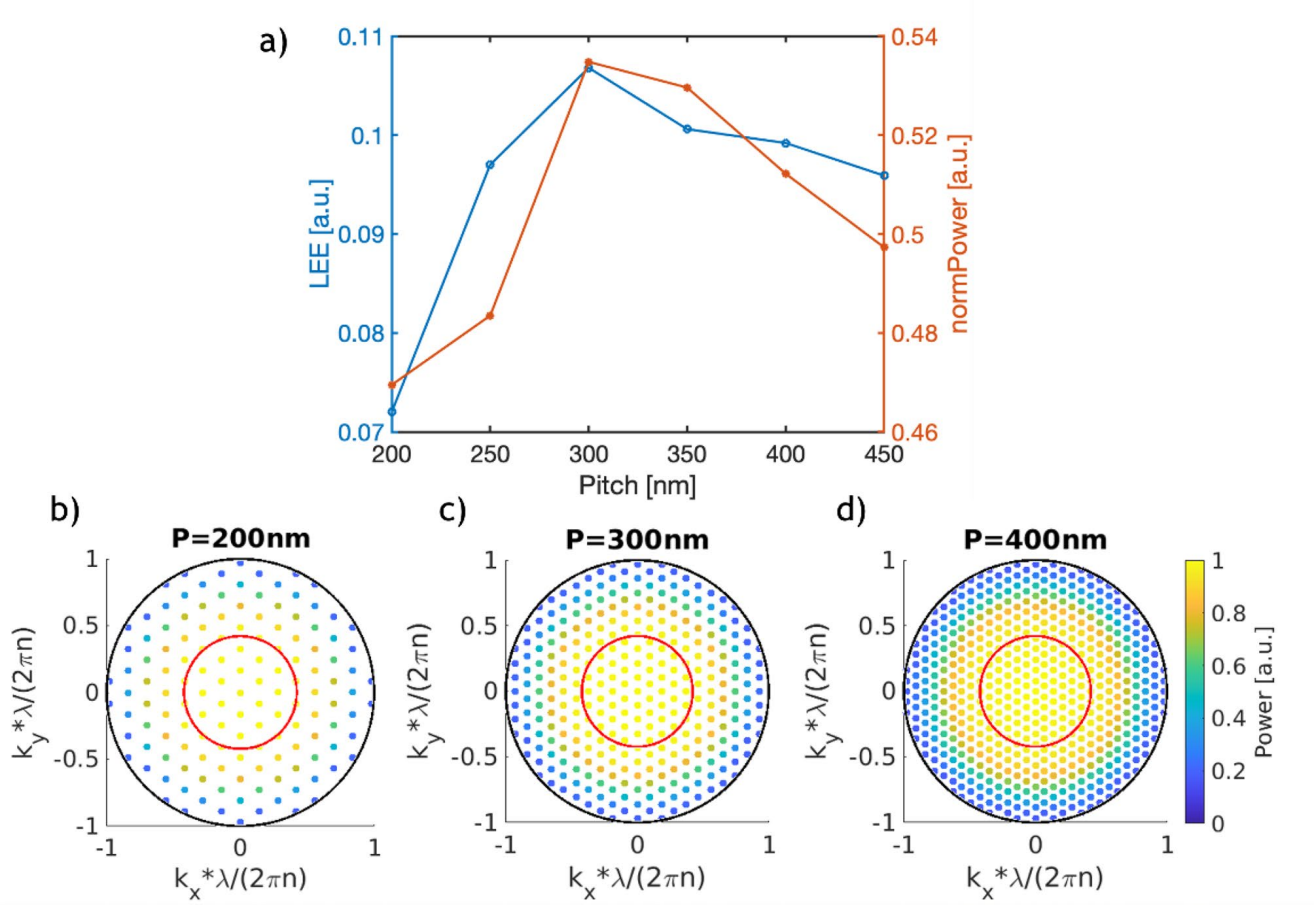
(**a**) LEE and normalized power emitter into the substrate as a function of the nanostructure pitch. (**b**–**d**) Normalized power distribution of modes emitted into the substrate for a pitch of 200 nm, 300 nm and 400 nm. The black outer circle represents the boundary of propagating modes, while the red inner circle represents the escape cone due to TIR.

The results of these parametric sweeps are presented in Figs. 2, 3, and 4.

The effect of varying the nanocylinder diameter on both the LEE and the power emitted into n-contact is shown in Fig. 2a. We see that both the LEE and emitted power rise non-linearly with increasing diameter, and that the LEE increases more relative to the emitted power. This led to the overall highest LEE observed of 15% for the 250 nm diameter nanocylinder. Noticeably, for this value of the diameter, the circular face of the cylinder is covering 63% of the area of the unit cell. This leaves just 37% for the p contact itself, which may have a detrimental effect on the p-contact conduction.

The dependence of the LEE on the diameter can be understood by decomposing the emission into plane waves, as in Fig. 2b–d for the cases of 50, 150 and 250 nm diameter. Each figure shows a set of colored points where the position refers to the transverse wave-vector components (k_x_ and k_y_) of the associated plane wave, and the color stands for the normalized power emitted in that mode (scale on the right side). The boundary between the propagating and evanescent spectrum, as well as the escape cone due to TIR are also shown. Every mode lying inside the escape cone has a propagation angle of less than 25° to the normal and therefore will contribute to the LEE. The critical angle can be determined via ${\theta }_{crit}=asin\left(\frac{{n}_{1}}{{n}_{2}}\right)$ where ${n}_{1}$ and ${n}_{2}$ are the refractive indices of air (1.0) and the n contact (2.37), respectively.

Figure 2b–d demonstrate that the larger the diameter, the more the emitted light is concentrated within the escape cone for emission to the exterior. Thus, increasing the diameter positively impacts the device, both by reducing p-contact absorption and also by concentrating the emitted light into the escape cone.

The LEE and emitted power are shown as a function of the nanostructure height in Fig. 3a. We see that even for very small values of the nanostructure height, the LEE increases significantly from 4.6 to 8.0% with respect to the planar reference. This suggests that even very shallow nanostructures have the potential to increase the reflection of the p-contact. Both the LEE and emitted power show an oscillatory behavior as a function of the nanocylinder height. In our simulations we used a fixed *z* position of the emitted dipoles (at the center of the MQW layer) while averaging over different *x–y* positions. Due to the fixed *z* position of the dipole, the position of the upper surface of the nanocylinder will produce constructive or destructive interference with the dipole emission depending on the distance between the two. This is the source of the oscillatory behavior. At the same time, the amplitude of the oscillations in the LEE and emitted power increase as the nanocylinder height increases. This can be understood as a consequence of the decrease in the amount of p-contact material that the light crosses before being reflected at the top interface of the nanocylinder.

With the largest height value investigated of 140 nm, we see the largest increase in LEE. Remarkably, at this point the nanocylinder has penetrated through the entire p-contact, meaning that it is just touching the electron blocking AlN layer, and no more increase is feasible. On the other hand, Fig. 3b–d reveals that the power distribution as a function of transverse wave vector shows little variation with the height (see the right scale bar). For each of the different height values shown, the angular distribution of the emission remains constant, with a great part of the emission lying outside the escape cone. This emphasizes the fact that we cannot tune the angular dependence of the emission spectrum using the height of the nanocylinder. Instead, the impact of a taller nanocylinder is to remove more of the absorbing p-contact material, thus leading to a less lossy and more reflective p-contact. This directly increases the power emitted into the n-contact.

The variation of the emitted power and the LEE with the pitch of the nanostructure is shown in Fig. 4a. While varying the pitch, the aspect ratio of the diameter to pitch was kept constant at 0.5. The LEE has a peak at a pitch of 300 nm, which is slightly larger than the emission wavelength of 265 nm. The LEE reduces rapidly for pitches smaller than the emission wavelength. At the same time, increasing the pitch to values above 300 nm shows a slow decrease in LEE. Figure 4b–d show a very similar distribution of the emitted light over the transverse wave-vector, suggesting that the change in LEE is not dependent on more or less light being emitted into the escape cone. Despite this, there is a dependency of the LEE on the pitch. A possible explanation for this is that the coupling between the dipole and the lateral modes of the nanostructure is maximized when the nanostructure has a pitch slightly larger than the emission wavelength. The dependency of the LEE on the nanostructure pitch suggests that it is a key parameter that needs to be carefully optimized to obtain high efficiency devices. A tradeoff must be achieved with the diameter and height dependencies, where a larger diameter and height generally lead to a higher theoretical LEE, while possibly having adverse effects on the electrical performance.

Once an optimal parameter range for both optical and electric functionality has been found, the feasibility of fabrication should be addressed. Production scale techniques for nanostructured p-contact, such as those presented here, are readily available, i.e., nanosphere lithography^44,45^, nano-imprint technology^46^, displacement Talbot lithography^47^, and laser interferometry^22^. Using these techniques, nano-patterns with lateral sizes down to 100 nm can eventually be fabricated.

**Figure 5:**
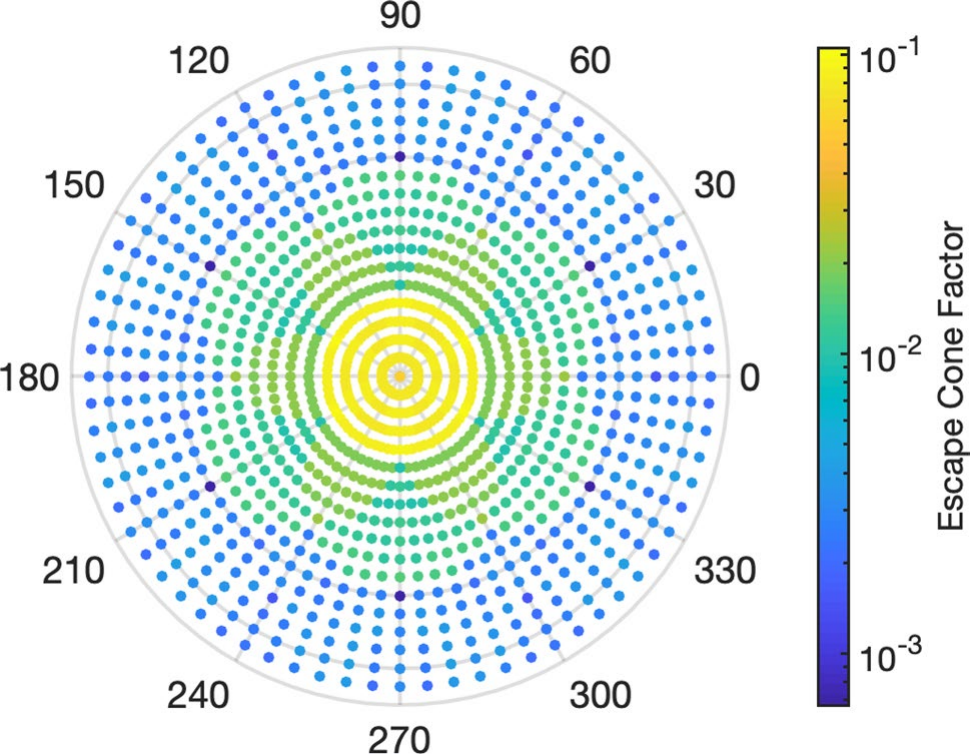
Normalized power scattered into the escape cone dependent on the incident angle of the light that has been reflected back from the substrate. See Fig. 1a. for description of escape cone and multiple passes through the device.

All of the results shown until now were made under the assumption that light coming back from TIR is lost. In order to verify this assumption, we have evaluated what we call the escape cone factor, which is shown in Fig. 5. We define this factor as the ratio of the light emitted into the escape cone to the power incident to the nanostructure returning from the substrate due to TIR. In order to compute this factor, we model plane waves incident to the nanostructure coming from the substrate side, instead of dipole sources. The incident angles of the plane waves are varied for polar angles *θ* from 0° to 85° and for azimuthal angles *φ* from 0° to 360°. We then compute the amount of reflected power that is contained inside the escape cone as a function of those angular ranges, with the results presented in Fig. 5. For the angular distribution of the light that is emitted into air see Supplementary Fig. S3.

For example, if we take a mode incident to the nanostructured p-contact with a polar angle of 30° and an azimuth of 0°, only around 2% of that incoming light will be scattered into the escape cone. The integration of the product of the escape cone factor and angular dependent reflection from the substrate due to TIR over all angles gives the additional LEE from a second pass through the device. This procedure can then be repeated for multiple passes using iteratively calculated values of the reflection from the substrate. Using this analysis, we estimate that the total LEE consisting of the original emission from the nanostructured region, plus two additional reflections of light due to TIR, results in an increase from 11.1 to 11.6%. This suggests that the light that is returning from the AlN/Sapphire and Sapphire/Air interfaces, once trapped via TIR, is not appreciably re-scattered into the escape cone of the DUV-LED device. Previous work on the optical benefit of patterned sapphire substrates (PSS) has shown that they have a small impact on LEE for low reflective p-contacts^48^. Since both the proposed nanostructure and PSS are able to extract trapped light, the combination of both concepts will provide an overall higher LEE than either concept in isolation. Despite this, the optimal case for exploiting PSS was shown to be a p-contact with very low losses, which is likely easier to obtain by using lower loss materials in a planar configuration.

## Conclusions

We presented an analysis of a nanostructured p-contact for integration in a deep-UV LED device operating at 265 nm wavelength. This analysis combines Brillouin zone and spatial integrations of rigorous simulations to obtain the response of quasi-isolated dipole sources in a periodic environment.

The proposed nanostructure consisted of a hexagonal periodic array of platinum nanocylinders embedded in the multi-layered p-contact. This was shown to reduce the absorption of the p-contact layer of the device, leading to an increase of the LEE from 4.6 to 11.1%. The LEE could be further optimized by increasing the diameter of the cylindrical nanostructure to 250 nm, which further concentrates the emission into the light escape cone. This leads to a LEE of 15.0% compared with the 4.6% of the planar reference. However, such large values of the diameter may lead to poor electrical performance. Therefore, the LEE of 11.1% for a diameter of 150 nm may be easier to realize.

Height variations revealed the importance of the penetration of platinum nanocylinder into the p-contact. The LEE rapidly increased from around 8 to 11% when the platinum nanocylinder height varied from 100 to 140 nm, corresponding to a change from partial to full penetration of the p-contact. It should be stressed that even with shallow nanostructures of only 20 nm height, the LEE was increased to 8.0%. This represents a large increase compared to the planar reference LEE of 4.6% while having a minimal impact on the device geometry.

The angular distribution of light emitted into the n-contact was not affected by the pitch variation, while keeping a fixed aspect ratio between the pitch and diameter. However, the total emission into the n-contact did vary and reached the highest for a pitch of 300 nm. This highlights the need to carefully optimize the pitch for proposed devices.

By analyzing the escape cone factor, we showed that multiple passes of light through the device had only a moderate effect on the LEE. For two additional passes through the device, the LEE increased from 11.1 to 11.6%. This suggests that, while the proposed nanostructure was able to reduce absorption in the p-contact, it was not able to significantly alleviate the problem of TIR. This could possibly be overcome by combining the nanostructured p-contact with other concepts for light extraction, such as nanopatterned sapphire substrates.

These results show that nanostructured p-contacts are capable of increasing the LEE of DUV-LEDs, in particular by reducing the absorption losses in the p-contact. Although the focus of this paper was exclusively the optical improvement, such optical gains should be carefully weighed against the potential reduction of the electrical performance due to the presence of the nanostructure. The limits of LEE enhancement via nanostructured p-contacts should be investigated by modelling a wide range of potential geometries, as well as using global optimization techniques to determine optimal parameters. In addition, direct modelling of the electric performance of the device should be performed for a complete description.

## Supplementary Information

Supplementary Information.

## Data Availability

The datasets generated during and/or analyzed during the current study are available from the corresponding author on reasonable request.
