# Supplementary material for: Tripling the light extraction efficiency of a deep ultraviolet LED using a nanostructured p-contact
Source: Sci Rep. 2022 Jul 7;12:11480. doi: 10.1038/s41598-022-15499-7 (PMC9262900; doi:10.1038/s41598-022-15499-7)
Supplement: Supplementary file 1 — Supplementary Information. [file 41598_2022_15499_MOESM1_ESM.pdf]

Supplementary Information: Tripling the light extraction efficiency of a deep ultraviolet LED using a nanostructured p-contact

Eduardo López-Fraguas[1], Felix Binkowski[2], Sven Burger[2,3], Sylvia Hagedorn[4], Braulio García-Cámara[1], Ricardo Vergaz[1], Christiane Becker[5], Phillip Manley*[2,3,5]

[1]*GDAF-UC3M, Dep. Tecnología Electrónica, Universidad Carlos III de Madrid. Avda. Universidad, 30. 28911, Leganés, Madrid, Spain*
[2]*Zuse Institute Berlin, Berlin, Takustraße 7, 14195 Berlin, Germany.*
[3]*JCMwave GmbH, Bolivarallee 22, 14050 Berlin, Germany.*
[4]*Ferdinand-Braun-Institut (FBH), Gustav-Kirchhoff-Str. 4, 12489 Berlin*
[5]*Helmholtz Zentrum Berlin für Materialen und Energie, Department Optics for Solar Energy, Kekuléstr. 5, 12489 Berlin, Germany.*
Contact information: phillip.manley@jcmwave.com

Figure S1 presents the intensity in the near field of the dipole source. In this case for the dipole situated at the center of the periodic unit cell. The geometrical parameters of the nanocylinder were for a pitch of 300 nm, diameter of 150 nm and height of 140 nm. Note that the results present in the main text include a further spatial integration over 64 positions in the *x-y* plane. Parts (a-b) show the intensity for a single Bloch Vector, $k_{Bloch} = (0,0)$. Interference fringes due to interaction with the neighboring dipole sources in nearby periodic unit cells can clearly be seen. In parts (c-d) the Brillouin Zone integration utilizes 16 Bloch vectors. In this case the interference fringes are strongly suppressed, thus approximating an ensemble of dipoles in the *x-y* plane emitting incoherently with respect to one another.

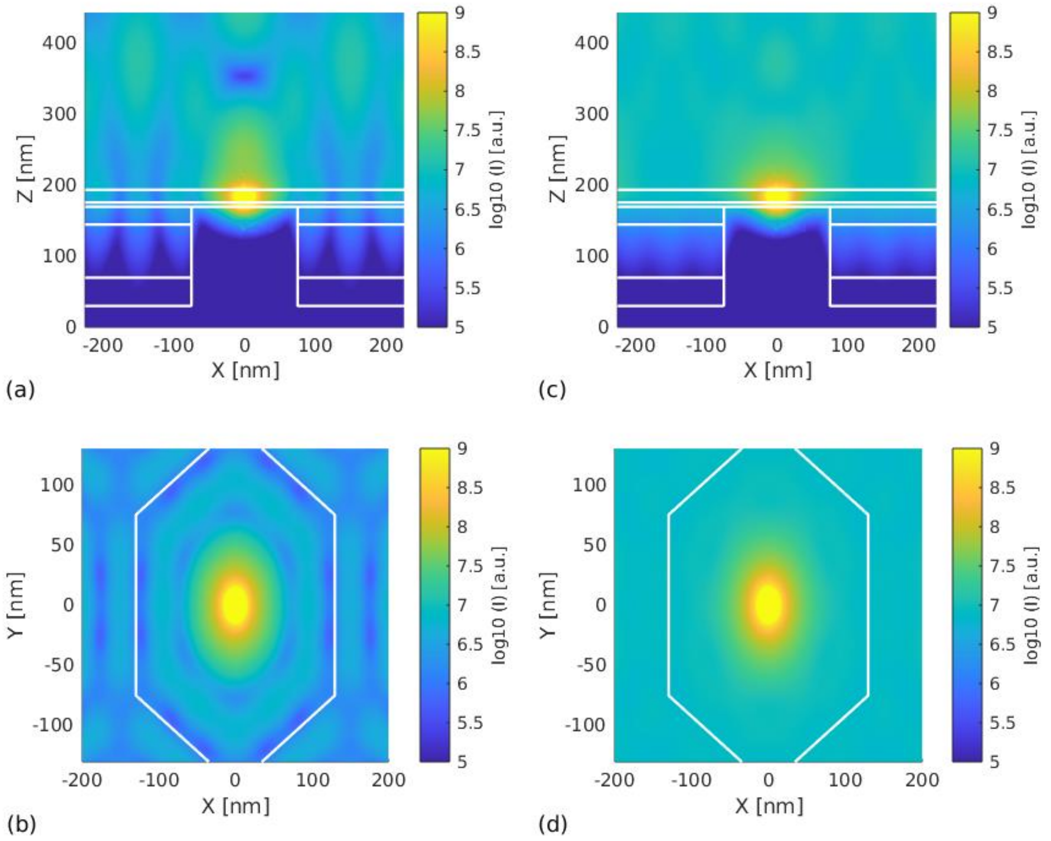

Figure S 1 The intensity in the near field of the champion device on a log scale. (a-b) The results obtained for a single Bloch vector in the Brillouin Zone integration. (c-d) The results obtained for the full Brillouin Zone integration of 16 Bloch vectors. White annotations indicate the geometry of the nanostructure and unit cell.

This work focuses on improving the emission of TE light from DUVLEDS. In fig S2 both the TE and TM LEE values are presented. Note the different scales in each case. Interestingly, the oscillations in the nanocylinder height dependence show the opposite phase for the TM polarized light.

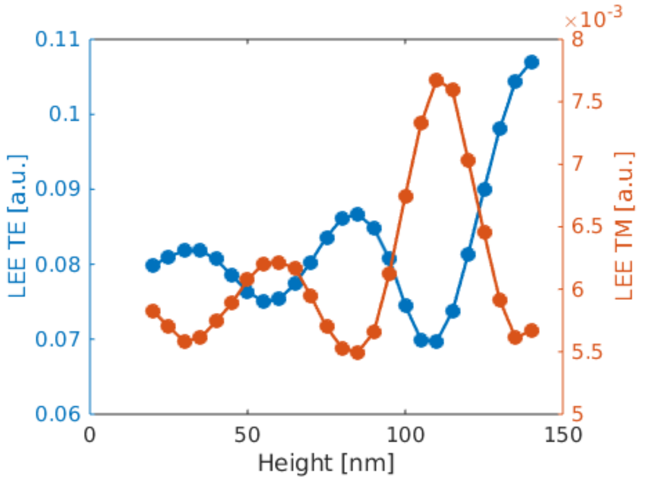

Figure S 2 The LEE of TE and TM emission as a function of the nanocylinder height. The values of the TE LEE refer to the left axis, while the TM LEE refers to the right axis.

Figure S3 presents the angular distribution of the light emitted from the device into air. Due to the periodic nature of the simulations, only a discrete set of angles are present. In the limit of increasing number of Bloch vectors used in the Brillouin Zone integration, this discrete distribution converges to a continuous distribution. The angular distribution contains exterior angles of up to around 65° in air. These high angles do not show a significant reduction in intensity compared to those at lower angles. It is expected that the intensity emitted into air will rapidly drop for higher angles as the emission inside the device gets closer to the total internal reflection boundary. The highest intensity is present at around 40° exterior angle.

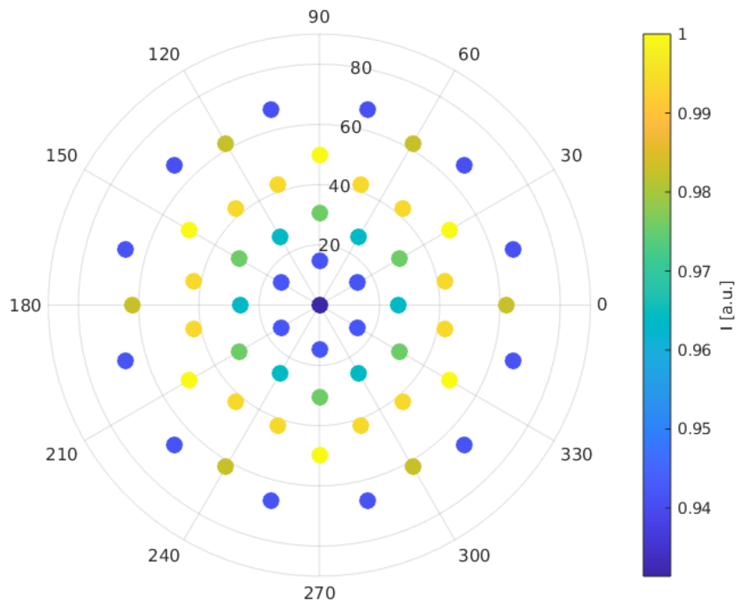

Figure S 3 The angular distribution of the light emitted from the device into air. Due to the periodic nature of the simulations, only a discrete set of angles are present. In the limit of increasing number of Bloch vectors used in the Brillouin Zone integration, this discrete distribution converges to a continuous distribution.
